# Sensory Modulation Abilities in Healthy Preterm-Born Children: An Observational Study Using the Sensory Processing and Self-Regulation Checklist (SPSRC)

**DOI:** 10.3390/biomedicines11082319

**Published:** 2023-08-21

**Authors:** Giulia Previtali, Cynthia Y. Y. Lai, Maria Valvassori Bolgè, Anna Cavallini, Renata Nacinovich, Daniele Piscitelli, Giulia Purpura

**Affiliations:** 1 School of Medicine and Surgery, University of Milano Bicocca, 20900 Monza, Italygiulia.purpura@unimib.it (G.P.); 2 Department of Rehabilitation Sciences, The Hong Kong Polytechnic University, Kowloon, Hong Kong SAR, China; 3 Department of Child Neuropsychiatry, IRCCS San Gerardo dei Tintori, 20900 Monza, Italy; 4 IRCCS Fondazione Don Carlo Gnocchi, 20148 Milan, Italy; 5 Doctor of Physical Therapy Program, Department of Kinesiology, University of Connecticut, Storrs, CT 06269, USA

**Keywords:** preterm, children, prematurity, sensory processing, sensory modulation, neurodevelopmental disorders

## Abstract

This study aimed to investigate prematurity as a risk factor for sensory processing disorders, using the Italian Version of Sensory Processing and Self-Regulation Checklist (SPSRC-IT), based on a sample of healthy Italian children born preterm in comparison with a sample of typical full-term children. Two groups of caregivers of Italian healthy preschooler children were recruited. The first group comprised 37 caregivers of full-term children (FT), while the second group consisted of 37 caregivers of preterm children (PT) (gestational age < 37 weeks). Significant differences between the groups in several subsections and factors of the SPSRC-IT were found, specifically in the Physiological Conditions section, in the Gustatory and Olfactory Sense section, in the Vestibular Sense section, and in the Proprioceptive Sense section, with lower scores in the PT group. Moreover, children born at a lower gestational age or with lower weights had a higher risk of dysfunctions in processing gustatory and olfactory, vestibular, and proprioceptive stimuli. In conclusion, the SPSRC-IT suggested a potential link between prematurity and challenges in the development of sensory processing and self-regulation skills, especially in children with a very low birth weight and very low gestational age.

## 1. Introduction

Sensory processing is a complex neurodevelopmental function allowing children to detect, modulate, perceive, discriminate, and integrate sensory inputs experienced from the environment or internally from their own body to effectively respond, learn, and adapt during daily life [1,2]. Both genetic and environmental factors play crucial roles in the early formation and fine-tuning of brain circuits necessary to receive, organize, and respond to sensory input, enabling individuals to behave in a meaningful and consistent manner [3]. Indeed, maturation of this function is very long and depends both on unimodal and cross-modal processes that are partially active but very immature at birth and that gradually develop through environmental experiences, playing an important role in some neurocognitive processes and behavior [4,5,6]. For these reasons, Dunn and collaborators suggested the importance of knowing these aspects to interpret children’s behaviors, and based on their studies, they hypothesized an important relationship between the neurological threshold of the individual (that depends on the ease or difficulty of activating the central nervous system (CNS) in response to sensory stimuli) and their self-regulation strategies (necessary to manage the amount of sensory inputs during the interaction with the world) [7]. Based on these issues, Dunn proposed the presence of strong relationships between sensory processing and temperament, as well as personality traits. This is because sensory processing preferences reflect the individual’s nervous system needs and underpin behavioral regulation, adaptive responses to novel challenges, and environmental adaptation [8]. Additionally, De Gangi and colleagues [9] conducted an in-depth study of the interlink between sensory processing and self-regulation. They suggested that children initially identified with dysfunctions in these neurodevelopmental domains during the first years of life are at a high risk of later perceptual, language, and emotional/behavioral difficulties in the preschool and school years.

Considering the emerging scientific evidence related to this topic, several authors described a group of early developmental conditions in which dysfunctions in sensory processing and integration abilities are evident and impact daily life, despite the absence of brain damage or primary sensory impairment [10,11,12]. Indeed, sensory processing disorders (SPDs) are included in the Diagnostic Classification of Mental Health and Developmental Disorders of Infancy and Early Childhood—DC: 0–5 [13] as a specific and distinct disorder from other psychopathological conditions, although problems in sensory domains may also occur concurrently with other neurodevelopmental disorders [10,14,15,16]. According to the DC:05, SPDs are a group of conditions in which, despite the integrity of the sense organs, the infant/young child demonstrates behaviors that are believed to reflect abnormalities in regulating sensory inputs, causing distress, or impairing functioning in daily activities. Typically, children with SPD have a constant need to re-regulate their senses to adapt to the stimuli around them, which creates symptoms of distractibility, irritability, anxiety, and depression. SPDs include Sensory-Over Responsivity Disorder (heightened magnitude of response, faster latency of response, slower habituation or recovery from the response to sensory stimuli), Sensory-Under Responsivity Disorder (reduced magnitude of response, slower latency to respond to sensory stimuli) and Other Sensory Processing Disorder (atypical response to stimuli and extended sensory exploration of stimuli, for example licking walls or doorknobs).

In fact, while the developmental trajectories and neural correlates of sensory processing and integration dysfunctions are not completely understood, the association between sensory processing sensitivity and various aspects of social functioning is becoming clearer nowadays. These early dysfunctions may interfere with participation in life situations [3]. In this context, some authors reported a high frequency of sensory processing dysfunctions in preterm-born children, confirming the adverse effects of these issues on regulatory, perceptual, and motor development [17]. Causes for this type of dysfunction in this population and in the absence of significant neurological impairments are unclear. Still, some relevant predictors for developing sensory modulation problems in preterm children were identified, including the gestational age, length of Neonatal Intensive Care Unit (NICU) stay, Apgar scores, and presence of white and/or grey matter brain abnormalities [18].

Indeed, it is widely recognized that despite advancements in perinatal medicine and enhanced quality of care in NICUs, preterm birth remains the primary cause of mortality in children under the age of 5. Furthermore, it is also the leading cause of motor disabilities, neurosensory impairments, developmental delays, cognitive challenges, and behavioral and language disorders, even in high-income countries [19].

Specifically, the incidence of atypical sensory profiles in preterm-born children seems to be between 39% and 52%, with some evidence suggesting that babies born before 32 weeks of gestational age have the highest risk [20,21,22].

Eeles and colleagues suggested that these dysfunctions may contribute to the reduced cognitive development, the atypical temperament and social-emotional problems, and language and motor outcomes of these children compared with their term-born peers [23,24].

Therefore, this study aimed to investigate prematurity as a risk factor for sensory processing disorders, using a new assessment tool, that is the Italian Version of Sensory Processing and Self-Regulation Checklist (SPSRC-IT) [25], based on a sample of healthy Italian preterm-born children in comparison with a sample of typical full-term children.

The decision to utilize the SPSRC for this investigation is supported by recent validation studies conducted in diverse cultural settings. These studies have demonstrated that the SPSRC is effective in assessing both the regulatory capacities and behavioral responses to sensory stimuli encountered by children in their daily activities providing interesting insight for clinical practice in childhood [25,26,27].

## 2. Materials and Methods

### 2.1. Sampling and Data Collection

Two groups of parents and caregivers of Italian children were recruited for this study. The first group comprised 37 parents/caregivers of children with typical development and full-term birth (FT). The second group consisted of 37 parents/caregivers of children with typical development and preterm birth (PT) (birth at gestational age < 37 weeks). Table 1 shows the distribution of the PT group’s sample according to gestational age. It is important to note that children diagnosed with developmental disorders, including neurological, psychiatric, or significant sensory impairments, were excluded from both groups. In detail, children with developmental disorders were excluded from the study by asking caregivers to report any diagnosis of developmental disorders. None of the included children participated in cognitive training or behavioral therapies. 

The sample was composed of 74 healthy preschooler children (34 M; 40 F) with a mean age of 4.5 years (SD: 1.0), divided into the two groups matched for age and sex. Table 2 displays the demographic data of the participants. The person who spends the most time with the child was asked to fill out the questionnaire (89.2% mother, 4.05% father, 1.35% both, and 5.4% others).

Participants were recruited through an official application to participate in the project and sent along with an information sheet that presented and explained the study. No incentive was given to the participant. The participation was on a voluntary basis. The questionnaire was filled out by the parents/caregivers using an online self-administered form with Google modules (Mountain View, CA, USA). Data were then stored in an MS Excel workbook. The whole compilation was completely anonymous. The study was conducted following the Declaration of Helsinki.

### 2.2. Sensory Processing and Self-Regulation Checklist

The Sensory Processing and Self-Regulation Checklist (SPSRC) is a questionnaire parent report designed by Lai and Chiu [28], administrated to the primary caregiver. The checklist provides sensory processing and self-regulation information for children aged 3 to 8 years and is based on Ayres’s sensory integration theory [29]. The Italian Version of the Sensory Processing and Self-Regulation Checklist (SPSRC-IT) was validated by Purpura and colleagues [25], demonstrating excellent psychometric properties. Moreover, good convergent validity with respect to other outcome measures (in particular Sensory Profile-2) was reported [25].

The SPSRC-IT comprises 125 items, divided into two parts, several sections, and factor scales. Part 1 (33 items) investigates self-regulation and has three sub-sections [(A) Physiological Conditions, (B) Social/Cognitive/Emotional Development, and (C) Behaviors When Facing Changes or Challenges] and four factor scales [(1) Emotional Regulation, Facing Challenges; (2) Emotional Regulation, Facing Changes; (3) Physiological Regularity and Response to Soothing; and (4) Autonomic Activity]. Part 2 (92 items) consists of items related to sensory processing and is further divided into six sub-sections [(A) Auditory Sense, (B) Visual Sense, (C) Tactile Sense, (D) Gustatory and Olfactory Sense, (E) Vestibular Sense, and (F) Proprioceptive Sense] and four factors [(1) Sensory-Seeking Behavior, (2) Sensory Under-Responsivity, (3) Sensory Over-Responsivity, and (4) Stability of Sensory Responsivity]. The caregivers were instructed to report on their child’s typical performance within the last three months for the items on the checklist using a 5-point Likert scale (5 = never, 4 = seldom, 3 = sometimes, 2 = frequently, and 1 = always); some items had opposite polarity (thus, a reversed scoring). A higher score indicated a more favorable performance (fewer negative symptoms). Table 3 shows example of some items from the SPSRC-IT.

### 2.3. Statistical Analysis

Anonymous data from the participants were initially encoded by MS Excel. Subsequently the statistical analysis was made possible using the JASP Version 0.17.2 [Computer software; University of Amsterdam; 2023]. A Shapiro–Wilk test for normality was performed. Since, within the two groups, there were variables with both normal and not-normal distributions, we decided to use a non-parametric approach.

A comparison of the FT group to the PT group for several scores of the parts, subscales, and factors of the SPSRC-IT was performed using a Mann–Whitney U test. Descriptive analyses were reported where appropriate. Moreover, a two-tailed bivariate non-parametric correlation test (Spearman Test, ρ) was performed between the mean scores of several sections and factors and the age of children. Finally, Spearman partial correlation analysis, controlled for the age of children, was performed between several mean scores of the SPSRC-IT and two clinical indexes at birth (gestational age and weight at birth). A *p*-value below 0.05 was set as significant.

## 3. Results

### 3.1. Differences between the Two Groups

Concerning the Total Scores and the Composite Scores of Parts 1 and 2 of the SPSRC-IT, FT children showed more favorable results compared to PT children (Total Score = FT mean: 568.2, SD: 30.3; PT mean: 548.5, SD: 39.1; Part 1 = FT mean: 143.1, SD: 8.9; PT mean: 138.6, SD: 12.7; Part 2 = FT mean: 425.1, SD: 24.4; PT mean: 409.9, SD: 30.03), although both groups showed scores in the range or near the range of the norm.

Significant differences in the Total Scores (*p* = 0.022; w = 896.5) and the Scores of Part 2 (sensory processing) (*p* = 0.024; w = 894.0) between the groups (Table 4) were observed. Moreover, significant differences were found in the following subscale sections: 1A—Physiological Conditions section (*p* = 0.008; w = 929.5), in the 2D—Gustatory and Olfactory Sense section (*p* = 0.012; w = 913.5), in the 2E—Vestibular Sense section (*p* = 0.011; w = 920.5), and in the 2F—Proprioceptive Sense (*p* = 0.031; w = 884.5), with lower scores in the PT group (Table 4). No significant differences were observed in the other subscales between the two groups. Finally, differences between the two groups were also found in Factor 2—Emotional Regulation, Facing Changes of Part 1 (*p* = 0.023; w = 893.0), in Factor 2—Sensory Under-Responsivity of Part 2 (*p* = 0.044; w = 870.5.0), in Factor 3—Sensory Over-Responsivity of Part 2 (*p* = 0.031; w = 884.5), and in Factor 4—Stability of Sensory Responsivity of Part 2 (*p* = 0.034; w = 874.5). The detailed results are presented in Table 4.

### 3.2. Correlations

**Figure 1:**
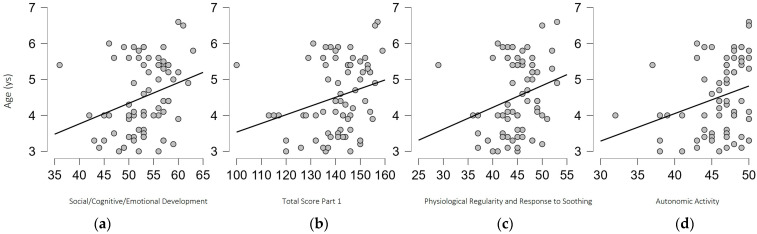
Graphic representation of the significant correlation between the age of children and (**a**) Social/Cognitive/Emotional Development Subscale, (**b**) Total Score Part 1, (**c**) Physiological Regularity and Response to Soothing Factor, (**d**) and Autonomic Activity Factor.

Considering the whole sample, results from the two-tailed Spearman’s correlation test highlighted the presence of a positive correlation between the age of children and the scores of 1B—Social/Cognitive/Emotional Development Section (ρ = 0.301, *p* = 0.009), the composite score of Score of Part 1 (ρ = 0.265, *p* = 0.022), the Factor 3—Physiological Regularity and Response to Soothing of Part 1 (ρ = 0.252, *p* = 0.030), and the Factor 4—Autonomic Activity of Part 1 (ρ = 0.269, *p* = 0.020), suggesting that higher scores in Self-Regulation are extremely linked to the normal growth and maturation of children (see Figure 1).

**Figure 2:**
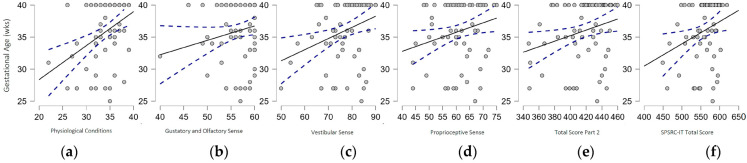
Graphic representation of significant correlation between gestational age and (**a**) Physiological Condition Subscale, (**b**) Gustative and Olfactory Sense Subscale, (**c**) Vestibular Sense Subscale, (**d**) Proprioceptive Sense Subscale, (**e**) Total Score Part 2, and (**f**) Total Score of the SPSRC-IT.

Successively, partial correlation analysis, controlled for age, showed a positive correlation between gestational age at birth and the 1A—Physiological Conditions section (ρ = 0.392, *p* < 0.001), the 2D—Gustatory and Olfactory Sense section (ρ = 0.288, *p* = 0.014), the 2E—Vestibular Sense section (ρ = 0.325, *p* = 0.005), the 2F—Proprioceptive Sense section (ρ = 0.274, *p* = 0.019), the Composite Score of Part 2 (ρ = 0.285, *p* = 0.015), and the Total score of the SPSRC-IT (ρ = 0.288, *p* = 0.014) (see Figure 2). Moreover, gestational age was positively correlated with Factor 1—Emotional Regulation, Facing Challenges and the Factor 2—Emotional Regulation, Facing Changes of Part 1 (F1: ρ = 0.287, *p* = 0.014; F2: ρ = 0.308, *p* = 0.008) and with Factor 2—Sensory Under-Responsivity and Factor 3—Sensory Over-Responsivity of Part 2 (F2: ρ = 0.294, *p* = 0.012; F3: ρ = 0.287, *p* = 0.014).

Finally, similar correlations were found between the weight at birth and the 1A—Physiological Conditions section (ρ = 0.333, *p* = 0.004), the 2D—Gustatory and Olfactory Sense section (ρ = 0.344, *p* = 0.003), the 2E—Vestibular Sense section (ρ = 0.295, *p* = 0.011), the 2F—Proprioceptive Sense section (ρ = 0.254, *p* = 0.030), the Composite Score of Part 2 (ρ = 0.293, *p* = 0.012), the Total score of the SPSRC-IT (ρ = 0.276, *p* = 0.018), Factor 2—Emotional Regulation, Facing Changes of Part 1 (ρ = 0.243, *p* = 0.038), Factor 2—Sensory Under-Responsivity, and Factor 3—Sensory Over-Responsivity of Part 2 (F2: ρ = 0.333, *p* = 0.004; F3: ρ = 0.274, *p* = 0.019).

## 4. Discussion

This study compared the abilities of sensory processing and self-regulation in healthy children born preterm and full-term, utilizing a new parent-report questionnaire, the Italian Version of the SPSRC. The primary result of the research study can be summarized as follows: healthy preterm children exhibit significantly lower abilities in certain domains of sensory modulation compared to full-term children, as well as in the absence of major sensory or neurological impairment, and these differences appear to be specifically associated with the degree of prematurity. While both groups showed similarities in the maturation of some self-regulation skills, children in the PT group displayed more difficulties in the modulation of gustatory/olfactory, vestibular, and proprioceptive stimuli compared to children in the FT group. This dysfunction is linked to neural messages that convey information and permit adjustments to the intensity, frequency, duration, complexity, and novelty of sensory stimuli. These results are in line with the findings of a recent review by Niutanen and colleagues [17], which examined the modalities to assess sensory functions in preterm children (from birth to nine years of age) and confirmed a variety of atypical responses to sensory modulation and somatosensory processing in this population. 

This aspect is crucial if we think that sensory processing dysfunctions are present in many neurodevelopmental disorders, such as autism spectrum disorders or others [10,12,14,15,30,31], but also that PT children have a higher risk of different neurodevelopmental disorders, also in the absence of brain damage [19,32,33,34]. In fact, several studies highlighted not only that the improved rates of survival in this population have been accompanied by increased rates of complications related to extremely preterm birth [35] but also that both late preterm and very preterm without brain damage, usually considered at a low risk of neurodevelopmental outcomes in comparison with children who are very preterm with brain abnormalities, actually have an increased risk of developing “minor” dysfunctions in some psychomotor and/or neuropsychological domains during preschool and school-age periods in comparison with full-term children [36]. For example, Brumbaugh et al. demonstrated that late PT school-aged children have more difficulty with processing speed, visual-spatial perception, and memory than their FT peers. Additionally, the authors reported that late PT children exhibited lower total brain tissue, increased cerebrospinal fluid, and smaller thalami in comparison to FT children. Therefore, the behavioral, cognitive, and structural findings also suggest that late PT birth may be considered a potential developmental challenge for the growing brain, given that these differences persist into school age [37].

In fact, there is evidence supporting the notion that preterm birth disrupts the typical trajectory of brain maturation. For these reasons, it is reasonable to consider that these neurodevelopmental dysfunctions are linked to the early and maladaptive extra-uterine experience in a period in which the CNS is particularly sensitive and vulnerable and not able to process and sustain the “bombardment” of sensory input from the surrounding environment [19].

Another example that confirmed difficulties on some domains of sensory processing in healthy PT children is linked to the known hypothesis of “vulnerability of dorsal stream”; as a matter of fact, while prematurity is not sufficient to compromise development of the visual ventral pathway, involved in object recognition according to size, shape, orientation, and color, maturation of the dorsal stream, which processes space and motion perception, is typically delayed in this population, also in absence of brain damage evident based on neuroimaging or in the absence of major sensory impairment, such as retinopathy of prematurity [38,39].

The second significant result derived from the partial correlation analysis, controlling for age, supports the growing literature: the gestational age and the weight at birth are strongly related to some sensory modulation and self-regulation abilities [21,22,40]. Specifically, in our study, children born at a lower gestational age or with a lower weight had a higher risk of physiological conditions, difficulties, and dysfunctions in processing gustatory and olfactory, vestibular, and proprioceptive stimuli. Also, these data are supported by other research, such as by the study of Rahkonen and colleagues [21] that highlighted that about half of the recruited preterm children born at an extremely low gestational age showed probable or definite atypical sensory profiles. 

These findings are plausible because premature birth abruptly disrupts the natural prenatal physiological maturation processes, which include the growth of the body’s organs and CNS, organization of the sleep–wake cycle, and sensory and motor systems. Instead, these processes are replaced by the highly medicalized artificial environment provided by modern incubators in NICUs. Consequently, also in the absence of brain damage, this situation may strongly affect the quantity and quality of early physical and social stimulation. In a period of high plasticity, atypical stimuli related to the environment (lights, sounds, materials) or to human relationships (physical contact, voice, eye contact) may have long-term repercussions on development and well-being and obviously, the lower the gestational age, the higher the possibility that this stressful situation can negatively influence brain development and the capacity of the child to adapt to the environment.

Regarding the relationship between the high prematurity and low levels of physiological conditions, this result is in line with data of the literature that suggest the presence of difficulties in this population based on executive functions and self-regulation abilities, which in turn appear to be related to poorer academic, behavioral, and adaptive functioning during school age [41,42].

Similarly, results of the correlation between sensory modulation and gestational age and birth weight can be associated with some vulnerabilities of preterm children during neurodevelopment.

For example, the processing of gustatory/olfactory stimuli could be associated with the frequent eating disorders of preterm infants and children. In fact, infants born preterm continue to experience high rates of oral-motor eating difficulties and behavioral eating challenges throughout the early developmental years, and in this population, the dietary patterns seem to be poor and often fall short of typical pediatric recommendations [43].

Regarding proprioceptive and vestibular processing, it is plausible to infer a connection between these sensory systems and the delayed maturation of motor abilities of preterm children during the preschool age [44] and the elevated prevalence of developmental coordination disorder (DCD) in preterm children [45].

According to van Hoorn et al. [46], preterm birth is a risk factor for DCD. Moreover, the existing evidence suggests that the DCD is often related to specific sensory disorders, and a recent review of Tran and collaborators [47] indicates a connection between proprioceptive deficits and motor impairment in DCD and that the proprioceptive status in the lower extremities predicted the balance ability in DCD.

These findings suggest the importance of the early monitoring of developmental trajectories, even in PT children without brain damage, as atypical sensory profiles in early childhood could serve as predictors of several difficulties during school age, ultimately impacting their quality of life. Indeed, it has been demonstrated that children with probable or definite differences in sensory processing experience significantly lower participation levels and enjoyment of participation compared to children with typical sensory processing abilities [9,48]. A few limitations should be acknowledged when interpreting the study findings. The study may suffer from recall bias because the parents/caregivers of preterm children may pay more attention to the sensory processing of their children [49]. However, the items of the SPSRC questionnaire specifically pertain to the children’s actual self-regulation and sensory processing. Secondly, as the data were collected through a customized self-reported questionnaire, it should be highlighted that no physiological assessments were recorded. Additionally, the SPSRC questionnaire is a Likert-based ordinal measure, which means that the precise interval between the scores is uncertain [50,51,52]. Future studies should apply Item Response theory models, such as Rasch Analysis, to provide raw-to-interval-score transformations [53,54,55]. 

## 5. Conclusions

In conclusion, the SPSRC-IT has proven to be a promising tool to highlight, along with the present literature, that prematurity harms the development of sensory processing and self-regulation skills, especially in newborns with a very low birth weight and very low gestational age. Finally, preterm children present significantly lower abilities in some domains of sensory modulation than full-term children, so the use of specific tools, such as the SPSRC-IT, can be fundamental for clinicians, therapists, and psychologists in order to identify and recognize these difficulties early and to program early and tailored interventions.

## Figures and Tables

**Table 1:** Summary of the sample of preterm-born children.

	Preterm Children (*n* = 37)
Extremely preterm babies (≤28 weeks GA)	9 (24.3%)
Very preterm babies (29–31 weeks GA)	4 (10.8%)
Moderate preterm babies (32–34 weeks GA)	10 (27.1%)
Late preterm babies (35–36 weeks GA)	14 (37.8%)

Abbreviation: GA, gestational age.

**Table 2:** Socio-demographic information of study participants.

	FT Group (*n* = 37)	PT Group (*n* = 37)
Sex (M; F)	17; 20	17; 20
Age (mean; SD)	4.5 (1.0)	4.5 (1.0)
Age range	3–6.6 years	3–6.5 years
Gestational Age (mean; SD)	39.8 (0.7) weeks	32.2 (3.6) weeks
Weight at birth (mean; SD)	3195 (406.8) g	1705 (649.9) g

Abbreviation: M, male; F, female, SD, standard deviation; g, grams.

**Table 3:** Example of some items of SPSRC-IT.

	English Items	Italian (Cross-Cultural-Adapted) Items [25]
**Part 1: Self-Regulation**		
Section 1A: physiological	Falls asleep easily at night (e.g., falls asleep after lying on bed within 20 min)	Si addormenta facilmente di notte (ad es. si addormenta dopo essersi sdraiato sul letto entro venti minuti).
Section 1B: social/cognitive/emotional	Unable to comprehend adults’ intentions or requests by observing their facial expressions, gestures, body languages, or speeches	È incapace di comprendere le intenzioni o le richieste degli adulti osservando il loro viso, espressioni, gesti, linguaggi del corpo o discorsi
Section 1C: facing changes or challenges	Throws temper tantrum or cries when he/she is asked to switch from one activity to another without advance notice during play or in family gathering	Quando gli viene chiesto di passare da un’attività ad un’altra senza preavviso mentre gioca o è in compagnia dei familiari fa i capricci o piange.
**Part 2: Sensory Processing**		
Scale 2A: Auditory	Appears excessively nervous, distressed, covers ears or complains about unexpected sounds (e.g., sounds produced by radio broadcast at MTR, alarm clocks or hand dryers)	Appare eccessivamente nervoso, angosciato, si copre le orecchie o si lamenta di suoni inaspettati (ad es. suoni della radio, sveglie o asciugamani ad emissione d’aria).
Scale 2B: Vision	Unable to notice or shows no response to flashing lights (e.g., neon lights or lights of Christmas decorations)	È incapace di notare o non mostra alcuna risposta alle luci lampeggianti (ad es. luci al neon o luci degli addobbi natalizi).
Scale 2C: Tactile	Appears excessively nervous, distressed or makes complaints while walking barefoot on a rough mat or grass mat	Quando cammina a piedi nudi su un tappeto ruvido o sull’erba appare eccessivamente nervoso, angosciato o si lamenta.
Scale 2D: Gustatory/Olfactory	Sniffs before manipulating objects or playing with toys	Annusa prima di manipolare oggetti o giocare con i giochi
Scale 2E: Vestibular	Unable to notice or shows no response when he/she is about to fall	È incapace di notare o non mostra alcuna risposta quando sta per cadere
Scale 2F: Proprioceptive	Likes to walk on tiptoes	Gli piace camminare in punta di piedi

**Table 4:** Comparison of the scores of the SPSRC-IT between the FT group and PT group (* *p* < 0.05, ** *p* < 0.01).

Scales/Factor	FT Group Mean (SD)	PT Group Mean (SD)	*p*-Value
**PART 1—Self Regulation**			
Section 1A: Physiological Condition	35.4 (2.9)	33.3 (3.8)	0.008 **
Section 1B: Social/Cognitive/Emotional Development	53.6 (4.5)	52.9 (5.6)	0.745
Section 1C: Behaviors When Facing Changes or Challenges	54.1 (4.0)	53.2 (4.7)	0.375
SPSRC-IT Score Part 1	143.1 (8.9)	138.6 (12.7)	0.119
Factor 1: Emotional Regulation–Facing Challenges	24.7 (3.2)	23.6 (3.4)	0.077
Factor 2: Emotional Regulation–Facing Changes	26.7 (2.2)	25.2 (2.7)	0.023 *
Factor 3: Physiological Regularity and Response to Soothing	45.4 (3.9)	44.5 (4.6)	0.621
Factor 4: Autonomic Activity	46.3 (3.2)	46.0 (3.9)	0.896
**PART 2—Sensory Processing**			
Section 2A: Auditory Sense	70.8 (4.7)	68.1 (6.1)	0.062
Section 2B: Vision Sense	63.1 (2.9)	62.5 (3.1)	0.331
Section 2C: Tactile Sense	90.6 (5.5)	89.4 (6.2)	0.582
Section 2D: Gustatory and Olfactory Sense	57.6 (3.5)	55.6 (4.3)	0.012 *
Section 2E: Vestibular Sense	79.4 (6.8)	74.5 (8.6)	0.011 *
Section 2F: Proprioceptive Sense	63.5 (7.3)	59.7 (7.7)	0.031 *
SPSRC-IT Score Part 2	425.1 (24.4)	409.9 (30.0)	0.024 *
Factor 1: Sensory Seeking Behavior	112.4 (5.6)	111.2 (5.6)	0.075
Factor 2: Sensory Under-Responsivity	139.6 (5.9)	135.9 (8.1)	0.044 *
Factor 3: Sensory Over-Responsivity	145.3 (16.2)	136.7 (16.9)	0.031 *
Factor 4: Stability of Sensory Responsivity	27.9 (3.2)	26.0 (4.4)	0.034 *
**SPSRC-IT Total Score**	568.2 (30.3)	548.5 (39.1)	0.022 *

## Data Availability

The data presented in this study are available on request from the corresponding author. The data are not publicly available due to ethical reasons.
